# The PI3K/AKT Pathway Inhibitor ISC-4 Induces Apoptosis and Inhibits Growth of Leukemia in Preclinical Models of Acute Myeloid Leukemia

**DOI:** 10.3389/fonc.2020.00393

**Published:** 2020-04-01

**Authors:** Charyguly Annageldiyev, Su-Fern Tan, Shreya Thakur, Pavan Kumar Dhanyamraju, Srinivasa R. Ramisetti, Preeti Bhadauria, Jacob Schick, Zheng Zeng, Varun Sharma, Wendy Dunton, Sinisa Dovat, Dhimant Desai, Hong Zheng, David J. Feith, Thomas P. Loughran, Shantu Amin, Arun K. Sharma, David Claxton, Arati Sharma

**Affiliations:** ^1^Division of Hematology and Oncology, Department of Medicine, Pennsylvania State University College of Medicine, Hershey, PA, United States; ^2^Penn State Cancer Institute, Pennsylvania State University College of Medicine, Hershey, PA, United States; ^3^Division of Hematology and Oncology, Department of Medicine, University of Virginia School of Medicine, Charlottesville, VA, United States; ^4^Department of Pharmacology, Pennsylvania State University College of Medicine, Hershey, PA, United States; ^5^Division of Hematology and Oncology, Department of Pediatrics, Pennsylvania State University College of Medicine, Hershey, PA, United States; ^6^Division of Hematology and Oncology, Department of Medicine, University of Virginia Cancer Center, Charlottesville, VA, United States

**Keywords:** AML, isoselenocyanate-4, Akt, apoptosis, AraC, xenograft, primary human AML cells

## Abstract

Acute myeloid leukemia is a heterogeneous disease with a 5-year survival rate of 28.3%, and current treatment options constrained by dose-limiting toxicities. One of the key signaling pathways known to be frequently activated and dysregulated in AML is PI3K/AKT. Its dysregulation is associated with aggressive cell growth and drug resistance. We investigated the activity of Phenybutyl isoselenocyanate (ISC-4) in primary cells obtained from newly diagnosed AML patients, diverse AML cell lines, and normal cord blood cells. ISC-4 significantly inhibited survival and clonogenicity of primary human AML cells without affecting normal cells. We demonstrated that ISC-4-mediated p-Akt inhibition caused apoptosis in primary AML (CD34^+^) stem cells and enhanced efficacy of cytarabine. ISC-4 impeded leukemia progression with improved overall survival in a syngeneic C1498 mouse model with no obvious toxic effects on normal myelopoiesis. In U937 xenograft model, bone marrow cells exhibited significant reduction in human CD45^+^ cells in ISC-4 (~87%) or AraC (~89%) monotherapy groups compared to control. Notably, combination treatment suppressed the leukemic infiltration significantly higher than the single-drug treatments (~94%). Together, the present findings suggest that ISC-4 might be a promising agent for AML treatment.

## Introduction

Acute Myeloid Leukemia (AML) is a highly heterogeneous malignancy. It is characterized by infiltration of abnormally and poorly differentiated hematopoietic cells in the bone marrow and peripheral blood. Despite recent achievements in AML drug discovery, the 5-year survival rate is 28.3% (1–3). Contemporary treatment regimens usually allow patients to enter remission, but the risk of relapse is still very high. AML arises from malignant transformation of immature hematopoietic stem and progenitor cells (HSPC) following an aberrant and poorly regulated leukemogenesis (4). The increasing frequency of Leukemic Stem Cells (LSC) has been shown at relapse after conventional chemotherapy contributing to increased drug-resistance (5). All this highlights that there is an urgent need for effective novel therapeutics for AML.

The PI3K/AKT/mTOR pathway is frequently upregulated in LSCs and plays a central role in multiple cellular functions, including survival, proliferation, apoptosis, cell cycle progression, and differentiation (6, 7). Aberrant activation of the PI3K/AKT has been reported in 50–80% of human AML (8). The activation of this pathway in AML occurs through multiple mechanisms, including signaling from NRAS, KRAS, KIT, FLT3-ITD, and other oncogenes signaling upstream of this pathway (9). Such signaling may also be activated via IGF-1 and VEGF, which are paracrine/autocrine growth factors. Microenvironmental signaling via adhesion molecules and chemokines also plays a role in pathway activation (9). Upon activation, Phosphoinositide 3-kinase (PI3K) leads to the production of the secondary messengers PtdIns-3,4-P2 (PI3,4P2) and PtdIns-3,4,5-P3 (PIP3), which bind to Akt and result in its activation (10). Activation of the PI3K-AKT pathway is crucial for the maintenance of undifferentiated pluripotent stem cells, and cell differentiation and apoptosis follow the down-regulation of this pathway (11). Dysregulated Akt activation has been shown to alter HSPC function and lead to the development of AML in murine bone marrow transplantation model expressing myristoylated AKT1 (12). Recent studies showed that therapies targeting the PI3K-AKT may have great potential in AML therapeutics (13).

Isothiocyanates are compounds with anti-cancer properties and occur naturally in cruciferous vegetables (14, 15). Phenybutyl isoselenocyanate (ISC-4) is an isosteric selenium analog of phenylbutyl isothiocyanate with selenium replaced for sulfur (16, 17). Previous studies indicate that the mode of action of isothiocyanates is via inhibiting the PI3 kinase pathway (18, 19). Various selenium compounds are effective chemopreventive agents and act by modulating Akt activity in a many cancers (17, 20–22). ISC-4 has been shown to exhibit low systemic toxicity and high anti-tumor activity in both *in vitro* and *in vivo* melanoma preclinical models (16, 17). Also, treatment with ISC-4 led to significant apoptosis in melanoma cells (17). Topical application of ISC-4 led to delayed development of melanocytic lesions in animals with invasive xenografted human melanoma (23). Studies on colon cancer showed that ISC-4, both as a single agent and in combination with the anti-EGFR monoclonal antibody cetuximab (24), led to increased apoptosis of cancer cells *in vitro* and *in vivo*.

In the present study, we report the anti-leukemia property of ISC-4 against AML cells *in vitro* and *in vivo*. We demonstrated that ISC-4-mediated p-Akt inhibition caused apoptosis in primary AML (CD34^+^) stem cells and enhanced efficacy of cytarabine (AraC). Besides this ISC-4 showed pre-clinical efficacy with a significant survival benefit in AML animal models. Altogether, our findings suggest that ISC-4 might be a potent agent for targeting patients with AML and pave the path for the future development of ISC-4 in combination with other agents.

## Materials and Methods

### Cell Lines

The human AML-derived cell lines HL-60, U937, MV4-11 and mouse leukemia cell line C1498 were obtained from the American Type Culture Collection (ATCC), Manassas, Virginia, USA. All other cell lines were kindly gifted according to the acknowledgments. All the cells except for MV4-11 cells were grown in RPMI 1640 culture medium (Corning, Manassas, VA) supplemented with 10% heat inactivated fetal bovine (FBS) (Atlanta Biologicals, Lawrenceville, GA) and 1% Penicillin and Streptomycin (GIBCO, Gaithersburg, MD). MV4-11 cells were cultured in the IMDM (Iscove's Modified Dulbecco's Medium) culture medium (Corning). Cell lines with low-passage stocks were used and cultured at 37°C and 5% CO2 in a humidified incubator for <2 months.

### AML Patient Samples

Bone marrow aspirates or peripheral blood samples were obtained from AML patients. Normal peripheral blood samples were obtained from healthy donors and cord blood samples were obtained from the freshly delivered placenta of the healthy donor after informed consent using protocols approved by the Penn State College of Medicine Institutional Review Board (IRB). Mononuclear cells (MNCs) were isolated by density gradient separation (Ficol-Paque, GE Healthcare Life Sciences, Pittsburgh, PA) and frozen for later use. Primary human AML and normal cells were cultured in the serum-free expansion medium (SFEM) (Stem Cell Technologies, Vancouver, BC, Canada) supplemented with recombinant human stem cell factor (SCF), Interleukin 3 (IL-3), FMS-like tyrosine kinase ligand (FLT3-L), Granulocyte colony-stimulating factor (G-CSF), and granulocyte-macrophage colony-stimulating factor (GM-CSF). All cytokines were purchased from Shenandoah Biotechnology, Warwick, PA. StemRegenin1 (SR1) (ChemieTek, Indianapolis, IN), and UM729 (Selleckchem, Houston, TX) were added to maintain the “stemness” of primary cells for stem cell assays (25). All cultures were supplemented with 1% Penicillin and Streptomycin and stored in a humidified 37°C incubator with 5% CO_2_.

### Compounds

ISC-4 was synthesized using our previously described method at Penn State College of Medicine Organic Synthesis Core (16). Purity of ISC-4 (≥97%) was quantified by analytical high performance liquid chromatography (HPLC) analysis. Cytarabine (AraC, NDC 61703-305-38), Azacitidine (Aza, NDC 63323-771-39), and Daunorubicin (DNR, NDC 0703-5233-11) were purchased from the Penn State Hershey Medical Center Pharmacy. AraC was stored at room temperature, DNR at 4°C, and Azacitidine at −80°C. Venetoclax was purchased from ChemieTek (Indianapolis). ISC-4, venetoclax and GDC-0941 were reconstituted in DMSO and aliqouts were stored at −20°C. GDC-0941 (catalog # S1065) was purchased from Selleck Chemicals and reconstituted in DMSO. Aliquots were stored in −80°C.

### Cell Viability/Proliferation Assay

Viability and IC_50_ of AML cells following treatment with compounds was measured using CellTiter 96® AQueous One Solution Cell Proliferation Assay (Promega, Madison, WI) or Muse Count & Viability Assay Kit (MilliporeSigma, Burlington, MA) according to manufacturer's protocol. Briefly AML cells were plated into a 96-well plate at a density of 4 × 10^4^ cells per well in 100 μL of media and treated either with increasing concentrations of ISC-4 (0.093–12 μM), cytarabine (AraC, 0.07–5 μM), or venetoclax (0.625–40 nM) singly or in combination for 24, 48, or 72 h. For time kinetics studies, cells were plated in 24-well plate and exposed to ISC-4 for 3, 6, 12, 24, and 48 h. The background absorbance (at 490 nm) was subtracted from the data and normalized to controls. IC_50_ values for each compound in respective cell lines were determined from three independent experiments using GraphPad Prism 6.0 software (GraphPad Software, CA).

### Cell Cycle Analysis

Cells were plated in 6-well plate and treated with increasing concentrations of ISC-4 (0.5–3 μM) for 24 h. Post-treatment 1 × 10^6^ cells were washed with phosphate-buffered saline (PBS) (Corning) and fixed in chilled 70% ethanol for at least 3 h. Cells were washed with PBS and resuspended in 200 μL of Muse Cell Cycle reagent (MilliporeSigma), incubated for 30 min at room temperature, and analyzed on Muse Cell Analyzer (MilliporeSigma).

### Colony-Forming Assay

Cryopreserved human AML patient or cord blood mononuclear cells were cultured in Human Methylcellulose Complete Media (R&D Systems, Minneapolis, MN) in triplicate in 12-well plates at a density of 0.1–2 × 10^5^ cells per well. The optimal plating densities were selected for each case to yield colony out-growth of 20–100 colonies per well. Human AML cell lines were cultured at a density of 250–500 cells per well in Human Methylcellulose Base Media (R&D Systems). Two approaches were used to test the clonogenic potential of cells treated with ISC-4 against clonogenicity, first, cells were cultured in the presence of increasing concentrations of ISC-4 or DMSO simultaneously in methylcellulose medium for 7–14 days. Second, cells were exposed to ISC-4 (1–10 μM) or DMSO for 24 h. Post-treatment, cells were washed, counted and plated in methylcellulose-containing medium to grow colonies. Colonies were propagated for 7–14 days, and blast colonies (>20 cells/colony) were counted in a blinded manner under the light microscope. Colonies were imaged with the Olumpys CKX31 inverted microscope (Olympus corporation, Center Valley, PA) using the 4X objective.

### Western Blotting

Cells were plated at a density of 0.5–1 × 10^6^ cells/mL in 6-well plates and treated with increasing concentrations of ISC-4 or controls, harvested at different time points (6, 12, or 24 h), and washed with cold PBS. Whole cell lysates were harvested in 100 μL 1X RIPA buffer (Sigma) containing phosphatase inhibitor cocktails 2 and 3 (Sigma) and protease inhibitor cocktail. Protein was quantified using the bicinchoninic acid (BCA) assay kit (Pierce, Thermo Fisher Scientific, Waltham, MA). Denatured protein samples were resolved on a NuPAGE 4–12% Bis-Tris gel (Life Technologies Carlsbad, CA) and transferred to PVDF membranes (Life Technologies). Membranes were blocked for 1 h at room temperature in 5% milk/TBS-T, incubated overnight at 4°C with primary antibodies (1:1,000), and immunodetection was done with corresponding secondary IgG HRP-linked antibodies (1:5,000) using the ECL chemiluminescence reagents (Thermo Fisher Scientific), and detected by Bio-Rad ChemiDoc MP imaging system (Bio-Rad Laboratories, Hercules, CA). Bands were quantified by Bio-Rad ImageLab 6.0.1 software. Antibodies against various proteins were obtained from the following sources: Akt, phospho-Akt (Ser473), PARP, caspase 3, cleaved caspase 3, caspase 9 (Cell Signaling, Danvers, MA); GAPDH, (Santa Cruz Biotechnology, Dallas, TX); The goat anti-rabbit IgG-horseradish peroxidase (HRP) conjugates and goat anti-mouse IgG-HRP conjugates were purchased from (Cell Signaling).

### Flow Cytometry

Apoptosis was assessed post-treatment with either ISC-4 or controls for the indicated time points and doses. Samples were stained using the Muse Annexin V & Dead Cell Kit (MilliporeSigma). Caspase activation was measured using the Muse Caspase-3/7 Assay Kit (MilliporeSigma). Multicaspase (caspase-1, 3, 4, 5, 6, 7, 8, and 9) was assayed by MultiCaspase Assay kit (MilliporeSigma), and MitoPotential kit (MilliporeSigma) was used to determine the percentages of cells with mitochondrial depolarization. All kits were used according to manufacturer's protocol. Cells were then analyzed by benchtop flow cytometer Muse Cell Analyzer (MilliporeSigma).

In order to detect the apoptosis in LSCs, cells were stained with anti-human CD45-APC-Cy7, and either with CD34-FITC, CD123-APC, or TIM-3-PE-Cy7 monoclonal antibodies for 30 min on ice, washed with staining buffer and stained with Annexin V-BV421 and 7AAD to detect apoptosis.To study the Akt phosphorylation in AML patient samples, cells were surface stained with CD45-APC-Cy7 and CD34-BV421 antibodies followed by intracellularly staining with p-Akt-PE (Ser473) antibody (Cell Signaling). Cell viability was assessed as described previously with a Fixable Viability Dye eFluor 660 (eBioscience, San Diego, CA) (26). Antibodies used for cord blood cells were CD45-APC-Cy5, CD34-FITC, CD38-APC, and CD123-PE. For normal PBMC cells CD45-APC-Cy5, CD3-FITC, CD19-APC, CD11b-PE, and 7AAD anitbodies were used. All the antibodies were purchased from BioLegend (Dedham, MA) unless indicated otherwise.

### Preclinical Therapeutic Efficacy of ISC-4 in Xenotransplantation Models of AML

A syngeneic C1498-dsRed-Luc and human xenograft U937 mouse models of AML were established as described previously (25). Cohorts of syngeneic mouse model were generated by transplanting luciferase-expressing mouse leukemia, C1498-dsRed-Luc cells into B6 (Cg)-Tyrc-2J/J (Albino B6) mice (The Jackson Laboratory, Bar Harbor, ME). When leukemia burden was clearly detectable by Bioluminescence Imaging (BLI), mice with equivalent bioluminescent signals were randomized into two treatment groups: vehicle control (DMSO) and ISC-4 (7 mg/kg). The whole-body leukemic burden was monitored by BLI throughout the study as described previously (25). The study was terminated, and all mice were euthanized when control mice displayed disease-related morbidity at day 27. A portion of the liver from control and treated groups were formalin-fixed and paraffin-embedded to examine changes in cell or organ morphology by Hematoxylin and eosin (H&E) staining. Images of gross liver morphology and stained section were captured using Nikon Eclipse Ts2R (Nikon, Melville, NY). The percentage of os-Red cells in the livers of mice was detected by flow cytometry. In the second set of C1498 animal experiment, Kaplan–Meier analysis was carried out to compare survival curves between vehicle control and ISC-4 (5 mg/kg)-treated mice.

For the human xenograft model, human AML U937 cells (1 x 10^4^) were engrafted by intravenous (I.V.) injection into 6-8-week old NOD.Cg-Rag1tm1MomIl2rgtm1WjlISzJ (NRG) mice (The Jackson Laboratory). Animals were randomized into treatment groups on day 5 based off of animal body weights. Beginning on day 6 (post-engraftment) mice were intraperitoneally (I.P.) injected with either vehicle control (DMSO), ISC-4 (7.5 mg/kg), AraC (50 mg/kg) or combination of ISC-4 and AraC every other day for 1 week. The study was terminated after the final treatment, and mice were sacrificed. Bone marrow cells were harvested, and leukemic tumor burden was evaluated by staining with APC-Cy7-labeled anti-human CD45 (BioLegend), mouse CD45-BV650 (BD Bioscience, Billerica, MA), and dead cell exclusion dye, 7AAD (BioLegend). Data were collected by flow cytometry using a BD LSR II flow cytometer. All of the animal procedures were approved by Penn State College of Medicine Institutional Animal Care and Use Committee (IACUC).

### Statistical Analysis

GraphPad Prism 6.0 software (GraphPad Software, CA) was used for the statistical analysis. Data were subjected to the Chou-Talalay method for determining the combination index using CalcuSyn software (Bio-Soft, Cambridge, United Kingdom). Combination index (CI) values plotted against fraction affected. CI values of <0.9 are considered synergistic, >1.1 are antagonistic, and values 0.9–1.1 are nearly additive. An unpaired *t*-test was used to determine the significance of differences between two groups, and one-way or two-way analysis of variance (ANOVA) was used to estimate the differences between three or more groups. All data are expressed as means ± Standard Error of the Mean (SEM) or Standard Deviation (SD) and are representative of at least two or three experiments. *P* < 0.05 (95% CI) are considered statistically significant.

## Results

### ISC-4 Induces Cell Proliferation in AML Cell Lines and Patient-Derived AML Blasts

**Figure 1:**
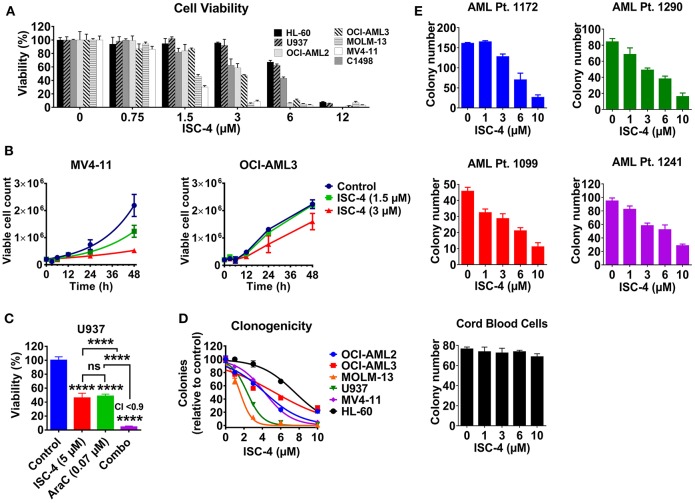
Effect of ISC-4 on AML cell proliferation. **(A)** Sensitivity of AML cell lines (*n* = 7) to ISC-4 (0.75–24 μM) after 24 h of treatment. **(B)** Inhibition of cell growth in MV4-11 and OCI-AML3 cells with ISC-4 treatment. **(C)** Effect of ISC-4 and cytarabine (AraC) combination treatment on U937 cell viability at 72 h **(D)** ISC-4-mediated reduction in clonogenicity of human AML cell lines in colony growth medium. **(E)** Sensitivity of primary human AML cells or cord blood mononuclear cells clonogenicity to ISC-4 treatment. Data are the mean ± standard deviation (SD) ****P < 0.0001; one-way ANOVA.

The effect of ISC-4 on AML cell viability was assessed in a mouse leukemia C1498 cells, and six human AML cell lines (MOLM-13, MV4-11, OCI-AML2, OCI-AML3, U937, and HL-60) with common genetic aberrations. Treatment with ISC-4 (0.75–24 μM) for 12 h inhibited cell proliferation indicating that ISC-4 indeed yields an overall antileukemia effect *in vitro* (Figure 1A). Half-maximal inhibitory concentration (IC_50_) values in the range of 2–7 μM (Table 1) revealed that, in general, MV4-11, MOLM-13, and OCI-AML2 were more sensitive than other cell lines tested. Furthermore, the cell growth of MV4-11 cells was found to be significantly inhibited by ISC-4 treatment with both concentrations at indicated time points (Figure 1B, left panel), extent of inhibition was less significant for OCI-AML3 cells (Figure 1B, right panel). These drug doses and time points were considered for the further experiments.

**Table 1:** IC_50_ values of ISC-4 for AML cell lines.

**Cell line**	**ISC-4 IC_**50**_(μM)**
MOLM-13	1.56 ± 0.52
MV4-11	1.57 ± 0.69
OCI-AML2	2.70 ± 0.63
OCI-AML3	4.15 ± 1.87
HL-60	6.91 ± 0.17
U937	6.22 ± 2.82
C1498	4.25 ± 0.39

Next, to test if ISC-4 could enhance the cytotoxic effect of cytarabine, an AML standard of care agent, OCI-AML3 and U937 cells were treated with increasing concentrations of ISC-4 (0.75–24 μM) and AraC (0.062–4 μM) simultaneously for 24, 48, or 72 h. It was very clear that the maximum combinatorial effect on the viability of both the cell lines was evident after 72 h drug exposure as shown in Figure 1C. The IC_50_ of AraC was found to be decreased by ~136-3,000-fold when cells were co-treated with 5 μM ISC-4 (Figure S1A). However, the combinatory effect was not significant at 24 or 48-h (Figure S1A). It is important to note here that AraC is a DNA-damaging agent and usually needs a longer incubation period to see cytotoxic effects on the cells (27–29).

To examine if ISC-4 would functionally inhibit the clonogenicity of AML *in vitro*, AML cell lines (*n* = 6) were exposed to ISC-4 (1–10 μM) for 7–10 days. A significant decrease in the number of colonies was observed compared to the control as illustrated in Figure 1D. As seen in the cell viability assay, yet again, a wide range of sensitivities was detected in response to the treatment.

Generally, cell lines are valuable scientific tools as they are highly proliferative and easy to culture. However, most of these cells lack various functional markers and may not represent the disease's original features (30, 31). Therefore, we extended our studies to primary human AML cells to validate the above observations. Primary human AML cases (*n* = 4) with various cytogenetic and molecular statuses (Table S1) were selected to test the effect of ISC-4 in cells capable of forming leukemic colonies. ISC-4 treatment resulted in a significantly reduced number and size of blast colonies (Figure 1E and Figure S1B).

Since ISC-4 inhibited cell proliferation and growth of AML cells as shown above, we were interested in examining whether ISC-4 would inhibit clonogenicity of progenitors in colony-forming assay. To study this, AML cells (OCI-AML3, U937, MV4-11, and AML Pt. 1172) were pretreated with ISC-4 (1–10 μM) for 24 h, washed and then cultured in a drug-free methylcellulose medium for 7–14 days to propagate the colony growth. Data revealed similar results as above with smaller and fewer colonies in ISC-4 treatment groups in a dose-dependent manner (Figure S1C). These data clearly suggest that ISC-4 does not only slow down cell proliferation, but it also inhibits the clonogenicity of blast progenitors.

Unfortunately, one of the major safety concerns for AML in the clinic is that conventional chemotherapy drugs also suppress the bone marrow, which is linked to the inhibition of HSPC proliferation (2). Cord blood is one of the best-known sources of HSPCs in the clinic (32). Therefore, to test whether ISC-4 exhibits dose-limiting toxicity in normal cells, we exposed CB-MNCs to ISC-4 in a colony-forming assay. Remarkably, ISC-4 did not affect the colony formation capacity of CB-MNCs from a healthy donor at the doses tested (Figure 1E). Besides the colony-forming assay, the toxicity of ISC-4 in premature cells defined by CD34^+^, CD34^+^CD38^−^ or CD123^+^ cells in cord blood samples, or normal T-cells (CD3^+^), B-cells (CD19^+^), and myeloid cells (CD11b^+^) in normal PBMC was also tested. CD34^+^, CD34^+^CD38^−^, or CD123^+^ cells were considerably more resistant to ISC-4 (Figure S2A), and indeed, minimal effects were seen on CD11b^+^ myeloid cells with ISC-4 up to 10 μM, suggesting a potential therapeutic window for the selective targeting of AML cells (Figure S2B). Thus, ISC-4 concentrations up to 10 μM were safely used in subsequent experiments. Overall, these results show that ISC-4 is active in AML, and able to inhibit the clonogenicity of leukemic cells, but not normal HSPCs, at the doses tested. Additionally, ISC-4 potentially can be combined with AraC to increase its potency against AML.

### ISC-4 Downregulates the Akt Signaling and Induces Caspase-Mediated Apoptosis

The PI3K/AKT pathway is frequently upregulated in AML and associated with poor prognosis (8, 33). Activation of Akt has been reported in ~70% of AML patients (8, 33). Inhibitors of this pathway have been shown to induce apoptosis in leukemia and other hematological malignancies (34, 35). Our group also previously reported that ISC-4 mediated downregulation of Akt pathway leads to apoptosis in melanoma, prostate and colon cancers (16, 17, 24, 36). Therefore, we sought to investigate whether ISC-4 could be used to target the Akt signaling in AML.

First, a time-course experiment was carried out to optimize the timing and dose of ISC-4. MV4-11 and OCI-AML3 cells were treated with different concentrations of ISC-4 (1.5 or 3 μM) up to 48 h. Apoptotic cell death (Annexin V positive) was observed in MV4-11 cells after 6 h of drug exposure, and continued to increase over time with a maximum at 48 h (Figure S3A). OCI-AML3 cells had similar time and dose-dependent response pattern with slightly less sensitivity toward ISC-4 (Figure S3A), which was consistent with previous experiments showing that MV4-11 cells are more sensitive than OCI-AML3 cells.

**Figure 2:**
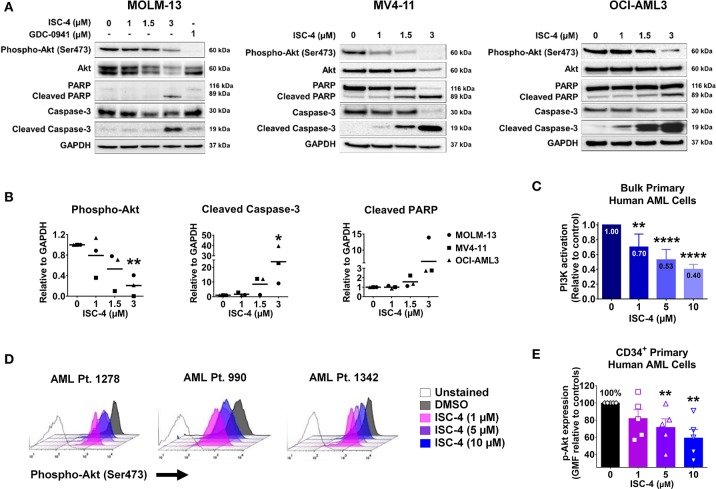
Inhibition of Akt signaling in AML by ISC-4. **(A)** Western blot analysis of AML cells exposed to increasing concentrations of ISC-4 or GDC-0941 (1 μM) for 24 h. GAPDH was used as a loading control. **(B)** Densitometric quantification of western blot bands. **(C)** Detection of PI3K activation by flow cytometry in bulk primary human AML cells (*n* = 3). **(D,E)** Flow cytometric detection of phospho-Akt (p-Akt) in CD34^+^ primary human AML cells (*n* = 5) after treatment with DMSO or ISC-4 (1–10 μM). Results are mean ± standard error of the mean (SEM). *P < 0.05, **P < 0.01, ****P < 0.0001 was assessed by one-way ANOVA.

Next, to determine if ISC-4 could downregulate Akt signaling in AML, cells were treated with increasing concentrations of ISC-4 (1–3 μM) for 6, 12 or 24 h. The ISC-4 concentration and exposure time were selected based on IC50 values and time-course studies. ISC-4 treatment for 6 h and 12 h led to a moderate decrease in Ser473 p-Akt levels in OCI-AML3 cells with no changes in total Akt levels (Figure S4A). However, decrease in Akt phosphorylation was significant after 24 h of ISC-4 treatment in all cell lines tested (Figure 2A). Treatment with a positive control GDC-0941, a selective PI3K inhibitor, also showed decreased Akt activity (Figure 2A). Interestingly, ISC-4 led to complete disappearance of p-Akt in the MV4-11 cells, instead, p-Akt in MOLM-13 and OCI-AML3 cells were still detectable at 24-h, represent residual p-Akt in AML (Figures 2A,B).

Time-course study of cells with ISC-4 treatment revealed an increase in activated caspases 9 and 3 (cleaved caspases 9 and 3) as early as 6 or 12 h following the treatment with ISC-4, but at relatively higher concentrations (3 μM) (Figure S4A). Cleaved caspase-3 was found to be significantly increased at 24 h time point even with the lowest concentration (1 μM) tested (Figure 2A). Activation of caspases 9 and 3 initiate the cleavage of cellular proteins including PARP. Our data showed a time and dose-dependent increase in cleaved PARP, a downstream of the caspase cascade, and marker of apoptosis. The time point of p-Akt inhibition correlated with the appearance of apoptosis (Figure 2A and Figure S4A). Densitometric quantification of western blots also clearly showed the downregulation of phosphorylated Akt and increase in cleaved caspase 3 and PARP (Figure 2B).

To further confirm the cell line data, ISC-4-mediated activation of the PI3K/AKT pathway was assessed in bulk primary human AML cells (*n* = 3). Cells were treated either with increasing concentrations of ISC-4 (1–10 μM) or a vehicle control (DMSO) for 24 h. ISC-4 treatment resulted in significant inhibition of PI3K/AKT activation in a dose-dependent manner in all cases tested (Figure 2C). Furthermore, alterations of p-Akt specifically in CD34^+^ primary human AML cells (*n* = 5) were also evaluated. Similar to the cell line results, flow cytometric analysis demonstrated a reduction in the p-Akt in CD34+ cells as depicted by a left shift in histograms (Figure 2D). Quantification of the data showed a significant decrease in the geometric mean of p-Akt-PE fluorescence in a dose-dependent manner (Figure 2E). These findings suggest that downregulation of Akt plays a functional role in the death of leukemia resulting from treatment with ISC-4.

**Figure 3:**
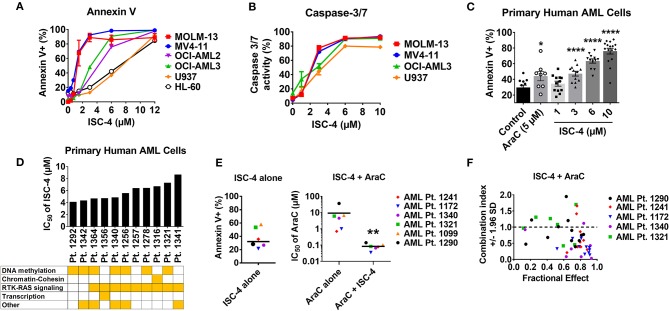
ISC-4 induces apoptosis in AML. **(A,B)** ISC-4-mediated apoptosis in human AML cell lines as the percentage of Annexin V^+^ or Caspase-3/7 activity. **(C)** Increase in Annexin V^+^ primary human AML cells after treatment (24 h) with ISC-4 (*n* = 11) or cytarabine (AraC, *n* = 8). Data are the mean ± SEM, *P < 0.05, ****P < 0.0001; one-way ANOVA**. (D)** IC_50_ determination for ISC-4 treatment of primary human AML cells with their genomic classification. **(E,F)** Enhancement of AraC treatment with ISC-4 in primary human AML cells (*n* = 6). **(E)** Efficacy of ISC-4 (5 μM) alone (left panel), AraC alone (5 μM) or the combination o in primary human AML cells (right panel). **(F)** Combination Index (CI) values for the ISC-4 and AraC combination treatment. Synergy CI <0.9. Data are the mean ± SEM, **P < 0.01, unpaired *t*-test.

To further extend our western blot outcomes, multiple cell lines were treated with increasing concentrations of ISC-4 (0.1–12 μM) for 24 h, and apoptosis was examined by flow cytometry. An increase in Annexin V^+^ cells indicates that early apoptosis could be detected within a low micromolar range (<5 μM) in most cell lines (Figure 3A). Additionally, flow cytometric analysis of Caspase3/7 and multicaspase activity was performed in OCI-AML3 cells (Figure 3B). The data show increased levels of various caspases including caspase-1,3,4,5,6,7, 8, and 9 with ISC-4 treatment (Figure S4B). One of the markers for apoptosis is release of mitochondrial cytochrome C which occurs due to mitochondrial membrane depolarization. We assessed this activity by treating OCI-AML3 cells with increasing concentrations of ISC-4 (1-10 μM) for 6 and 24 h. The data demonstrate that with increasing concentrations of ISC-4 there is an increase in number of cells with depolarized mitochondrial membrane (Figure S4C). Finally, pro-apoptotic potency of ISC-4 was supported by cell-cycle analysis showing an increase in the sub-G1 cell fraction in both MOLM-13 and MV4-11 cells in a dose-dependent manner (Figure S5). Overall, these findings suggest that ISC-4-mediated apoptosis could be the result of the intrinsic mitochondrial pathway activated by caspases-9 and 3.

In addition, combinations of ISC-4 with other standard AML drugs, daunorubicin (DNR), azacitidine (Aza) or venetoclax (VEN) were also tested in AML cells (MV4-11, OCI-AML3, and U937). Response of single-drug treatments was determined to select the concentration range to be used in the combination. The concentration of drugs was selected based on sensitivity of cell lines. Venetoclax: 5 nM for MV4-11, 1 μM for OCI-AML3 and U937 cells. ISC-4: 2 μM for OCI-AML3 and U937 cells, 0.5 μM for MV4-11 cells. Daunorubicin: 50 nM for all cell lines. Azacitidine: 2 μM for cell lines. ISC-4 enhanced pro-apoptotic activity of standard AML drugs in combination (Figure S6), suggesting the possible benefit of combining ISC-4 with the standard of care agents for AML.

### ISC-4 Induces Apoptosis in Primary Human AML Cells and Enhances Conventional Chemotherapy's Effects

Following the observation of ISC-4-induced apoptosis in cell lines, we wanted to validate these findings in primary human AML cells obtained from untreated AML patients (*n* = 11). The characteristics of the 11 patients with AML are shown in Table S1. Cells were treated with vehicle control, ISC-4, AraC or combo, and apoptosis was detected with Annexin V/7AAD staining as described previously (25). ISC-4 induced significant apoptosis in a dose-dependent manner with 3, 6, and 10 μM ISC-4 concentrations (Figure 3C). The effect of ISC-4 at a concentration between 1 and 3 μM was found to be comparable to 5 μM of AraC treatment (Figure 3C). The sensitivity of AML patient samples to ISC-4 was evaluated and cases were classified according to cytogenetic and molecular profiling as defined by Papaemmanuil et al. (37) (Figure 3D). We explored whether sensitivity to ISC-4 correlates with the mutational status of primary AML samples. No correlation was found between ISC-4 sensitivity and genomic classification by *NPM1, FLT3-ITD, DNMT3A, TET2, NRAS*, or *IDH1* mutational status (Figure S7). Next, ISC-4 (5 μM, close to the IC_50_ for primary cells) was combined with AraC (0.1–100 μM). Strikingly, ISC-4 reduced the IC_50_ of AraC by a median of ~66-fold (*P* < 0.01) (Figure 3E). Furthermore, the combination of ISC-4 (5–10 μM) and AraC (0.1–100 μM) in primary human AML cells (*n* = 5) resulted in combination index values lower than 0.9 in all cases tested indicating the synergism (Figure 3F).

### ISC-4 Induces Apoptosis in Primary Human AML Stem Cells

**Figure 4:**
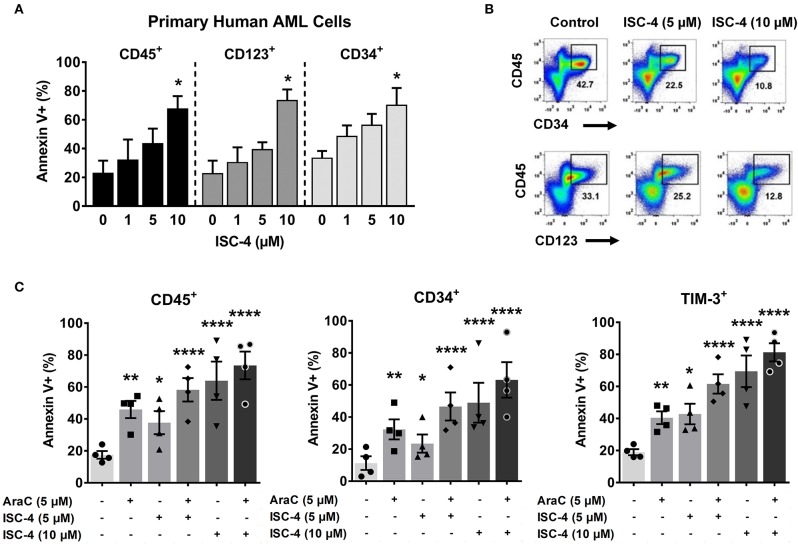
ISC-4 induces apoptosis in primary human leukemic stem cells. **(A)** Dose-dependent apoptotic response of bulk primary human cells (CD45^+^), or leukemic stem cells (CD34^+^ or CD123^+^ cells) to ISC-4. Error bars are mean ± SEM. **(B)** Reduction in leukemic stem cells after ISC-4 treatment in AML Pt. 1172. **(C)** Apoptosis in CD45^+^, CD34^+^, or TIM-3^+^ cells after ISC-4 and cytarabine (AraC) combination treatment. Error bars are mean ± SEM. *P < 0.05, **P < 0.01, and ****P < 0.0001; one-way ANOVA.

Since Akt regulates cell survival and apoptosis, and p-Akt is highly expressed in leukemic stem cells (LSCs) (7, 12), we investigated whether ISC-4 can induce apoptosis in LSCs. To study this, primary human AML cells were treated with increasing concentrations of ISC-4 (1–10 μM) for 24 h under the LSC-preserved culture conditions as described by Pabst et al. (38). Although a consistent immunophenotype of LSCs has not been identified, here we define them based off of their cell surface markers CD34, CD123, and TIM-3. ISC-4 induced apoptosis in both CD34^+^ and CD123^+^ LSCs in a dose-dependent manner, as shown in Figure 4A. ISC-4 also diminished the total volume of LSCs in AML Pt. 1172 (Figure 4B). Because we hypothesize that AraC may benefit from combination with ISC-4, AML cells were treated with single drugs or the combination. We found that ISC-4 enhances the pro-apoptotic activity of AraC in CD45^+^, CD34^+^, and TIM-3^+^ LSCs (Figure 4C). Here we have similarly shown that ISC-4 could induce apoptosis in TIM-3^+^ cells, comparable to the effect seen in CD34^+^ and CD123^+^ cells. These results show that ISC-4 induces apoptosis not only in bulk human AML cells but also in CD34^+^, CD123^+^, and TIM-3^+^ leukemic stem cells. Additionally, it has the potential to enhance the activity of AraC in AML stem cells, given that most of the cells were more sensitive to the combination compared to single-agent treatments.

### ISC-4 Reduces Leukemia Progression and Prolongs Survival in the Syngeneic C1498 AML Mouse Model

**Figure 5:**
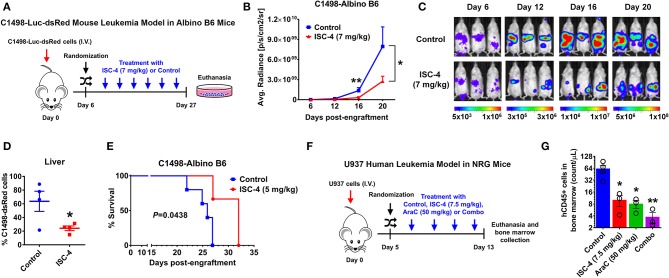
ISC-4 inhibits leukemia progression *in vivo* and extends overall survival. **(A–D)** Albino B6 mice (*n* = 3) transplanted with Luciferase and dsRed-expressing C1498 (C1498-dsRed-Luc) were treated either with vehicle control (DMSO) or ISC-4 (7 mg/kg). **(A)** Experimental scheme of C1498 animal study. **(B,C)** The decrease in the leukemic burden of ISC-4-treated mice monitored by bioluminescence imaging over the time course of the study. Data are mean ± SEM, *P < 0.05 analyzed by unpaired *t* test. **(D)** Flow cytometric analysis leukemic cells in liver harvested at the termination of the study. Data are mean ± SEM, *P < 0.05 analyzed by unpaired *t* test. **(E)** Kaplan-Meier survival analysis of C1498-bearing animals (*n* = 3–4) treated either with vehicle control (DMSO) or ISC-4 (5 mg/kg). **(F,G)** U937-bearing NRG mice (*n* = 3–4) were treated either with vehicle control (DMSO), ISC-4 (7.5 mg/kg), Cytarabine (AraC, 50 mg/kg) or combo. **(F)** Experimental scheme of U937 animal study. **(G)** Data shows a reduction of human CD45^+^ cells in the bone marrow of ISC-4, AraC, or combo-treated mice at the termination (day 13 post-engraftment) of study. Results are mean ± SEM; *P < 0.05, **P < 0.01; analyzed by one-way ANOVA.

Preclinical efficacy of ISC-4 in various disseminated AML mouse models was evaluated. First, ISC-4 was tested as a monotherapy in a syngeneic C1498 mouse model. Albino B6 mice with equivalent bioluminescent signals were randomized into two treatment groups: vehicle control (DMSO) and ISC-4 (7 mg/kg). ISC-4 was administrated by intraperitoneal (I.P.) route every other day following the treatment regimen as illustrated in Figure 5A. Analysis of BLI revealed ~3.2-fold decrease in the progression of leukemia after three doses of ISC-4 (day 12 post-engraftment), ~4.3-fold after five doses (day 16), and ~2.8-fold at day 20 (Figures 5B,C). The study was terminated, and all mice were euthanized when control mice displayed disease-related morbidity at day 27. Since in C1498 mouse model, leukemic cells primarily infiltrate the liver, a single-cell suspension was obtained by mechanical dissociation of liver at termination. The percentage of C1498-ds-Red cells in the liver of mice treated with ISC-4 was reduced by ~61% compared to the vehicle (*P* < 0.05) (Figure 5D). Gross anatomic examination of livers showed leukemic infiltrations as evidenced by the presence of several large chloromas (white arrows) in liver from vehicle-treated mice (Figure S8A). Conversely, ISC-4-treated mice livers had none to very few small residual chloromas (Figure S8A). Hematoxylin and Eosin-stained liver histology from the control-treated mouse revealed hepatic infiltration of leukemic cells, largely perivascular, whereas only minimal scattered nests of leukemia cells in the liver of ISC-4-treated mouse were observed (Figure S8B). Continuous treatment of mice with ISC-4 (7 mg/kg, every other day) for 20 days was well-tolerated with minimal weight loss (Figure S9A). Moreover, unlike conventional chemotherapeutics, continuous ISC-4 treatment did not cause noteworthy bone marrow suppression as evidenced by no significant changes in complete blood count (CBC) parameters (Figure S9B). These findings show that ISC-4 inhibits *in vivo* leukemia growth with negligible adverse effects.

Next, to test if ISC-4 treatment provides any overall survival benefit in animals, albino B6 mice engrafted with C1498 were treated either with ISC-4 (5 mg/kg) or a vehicle control every other day for 2 weeks. ISC-4-treated mice survived significantly longer (median survival, 32 days) (*P* = 0.0438) compared to control mice (median survival, 26 days) (Figure 5E). Hence, we conclude that ISC-4 can potentially extend the overall survival of animals.

### Antileukemic Activity of ISC-4 Combined With Cytarabine in a Disseminated Human AML Model

Next, we assessed whether ISC-4 has similar *in vivo* efficacy in a highly aggressive human AML xenograft model. The human AML cell line U937 was transplanted into NRG mice and animals were randomized into four treatment cohorts; vehicle control (DMSO), ISC-4 (7.5 mg/kg), AraC (50 mg/kg) or combination. Cohorts were treated with a total of 4 doses, as illustrated in Figure 5F, and study was terminated on day 13. Flow cytometric analysis of bone marrow cells exhibited significant reduction in human CD45^+^ cells in ISC-4 (~87% reduction, *P* < 0.05) or AraC (~89% reduction, *P* < 0.05) monotherapy groups compared to control (Figure 5G). Notably, combination treatment suppressed the leukemic infiltration significantly more than the single-drug treatments (~94%, *P* < 0.01) (Figure 5G).

Altogether, these studies suggest that ISC-4 exhibits preclinical efficacy in AML mouse models, and enhances AraC efficacy.

## Discussion

AML is a highly aggressive and heterogeneous hematologic malignancy characterized by the abnormal proliferation and differentiation of myeloid stem cells (1). The principal obstacle in AML treatment is relapse from complete remission (39). Mutations in LSCs confer resistance to chemotherapeutic agents and are the reason for relapse (40). As LSCs are accountable for tumor initiation, growth, and relapse in AML (39, 41–43), it is crucial to eliminate these LSCs for the long term survival of AML patients (44–46).

In the present study, we investigated the efficacy of ISC-4 as a potential chemotherapeutic agent in the treatment of AML. ISC-4 has been previously shown to be active vs. other neoplasms (16, 17, 24, 36). Our initial results in cell lines also showed ISC-4 as a promising agent for AML treatment. In cancer, the combinatory treatment approach yields better efficacy than monotherapy (47–49). Therefore, we tested ISC-4 along with the standard of care drugs daunorubicin, azacitidine, venetoclax and cytarabine. ISC-4 enhanced the effect of daunorubicin, azacitidine, venetoclax and cytarabine by increasing cell death. Since current intensive therapy is mostly AraC based, we pursued further combinatory experiments with AraC only. Furthermore, treatment with ISC-4 alone strongly inhibited the clonogenic potential of AML cell lines in a colony-forming assay. Subsequently, primary human AML cells were employed as they simulate the disease more closely than cell lines (30, 31). Once more, exposure to ISC-4 led to a marked decrease in blast colonies corroborating our previous cell line data. Bone marrow suppression is a common and undesirable side effect of many conventional chemotherapeutics (2). Our colony-forming data with normal CB-MNCs showed minimal inhibition effects, indicating that ISC-4 targets leukemic progenitor cells while sparing normal progenitor cells. An important point which is worth mentioning here is that we noted lower IC_50_ values of ISC-4 in AML as compared to previous reports in melanoma, colon, and prostate cancer (16, 17, 24, 36). Lower IC_50_ values signify higher drug activity for AML cells, in this case.

Multiple studies implicating the PI3K/AKT pathway in AML have been reported previously (50–52). Activation of this pathway, as identified by phosphorylation of the Ser473 residue on Akt, has been noted in ~ 70% of human AML (8, 33). A recent study demonstrated the role of the PI3K/AKT pathway in regulating autophagy and apoptosis in U937 cells (53). In a non-randomized phase-I clinical trial (NCT01396499) Buparlisib (BKM120), a pan PI3K inhibitor was tested on patients with refractory or relapsed acute lymphoblastic leukemia (ALL), acute myeloid leukemia (AML), or mixed phenotype acute leukemia (MPAL) who were non-suitable for standard chemotherapy. The study trial concluded that treatment with 80 mg/day of Buparlisib was tolerated well with modest single-agent efficacy and suggest its further evaluation in combination therapy in advanced hematologic malignancies (54). Inhibition of PI3K/AKT pathway has been shown to cause apoptotic cell death in AML (50).

Collectively, these data make Akt inhibition an attractive target for novel AML therapeutics. Our western blot results showed a significant decrease in the levels of p-Akt^Ser473^ upon inhibition with ISC-4, and we were also able to show increased cleaved PARP and caspase-3 levels following Akt inhibition. We also confirmed these observations by flow cytometry in AML cell lines and primary human AML cells which corroborate our western blot results. An important observation is that, in a limited group of primary human samples, we did not see any correlation between ISC-4 sensitivity and type of mutation present in primary AML samples. The ISC-4 and AraC combination studies enhanced the pro-apoptotic effect of AraC. It also displayed synergistic combination index values demonstrating the plausible advantage of combining ISC-4 with AraC. All the data provided here indicate that ISC-4 mediated inhibition of the Akt pathway induced apoptosis in AML.

Given the above data, we were interested to know the effect of ISC-4 on LSCs. It has been previously demonstrated that LSCs in AML are found within CD123^+^, CD96^+^, or TIM-3^+^ cellular fractions (25, 55–59). Therefore, we focused our experiments on these cell populations. Primary human AML cells treated with ISC-4 led to induction of apoptosis in both CD34^+^ and CD123^+^ LSCs. In our combination studies, we noticed that ISC-4 enhanced the pro-apoptotic property of AraC in CD45^+^, CD34^+^, and TIM-3^+^ LSCs. Altogether, these data provides an evidence that ISC-4 induces apoptosis in LSCs and enhances the anti-LSC activity of AraC.

Finally, our animal data provide clear evidence of ISC-4's efficacy in AML mouse models. Albino B6 mice transplanted with luciferase-expressing mouse leukemia, treated with ISC-4, displayed a slowed AML progression. Anatomic examination of livers showed leukemic cell infiltration defined by the presence of multiple large chloromas in vehicle-treated mice, whereas ISC-4 treated mice displayed very few small chloromas. Importantly, in our hands, continuous ISC-4 treatment did not lead to a notable suppression of bone marrow represented by unaltered complete blood count (CBC). These data suggest that ISC-4 inhibits *in-vivo* leukemia growth with minimal side effects and extends overall survival of animals. Modifications to ISC-4 treatment must be made to achieve superior results *in-vivo*. However, in a disseminated human AML model, combination therapy of ISC-4 and AraC suppressed infiltration of 94% of leukemic cells and was superior to monotherapies. These animal studies demonstrate that ISC-4 has a promising preclinical efficacy with a potential to be combined with current standard treatments for AML. Taken together, our data illustrate the anti-leukemic potential of ISC-4 and its promise as an AML therapeutic. Further modifications and clinically viable formulations of ISC-4 might significantly boost its potency and prove more active in AML clinical trials.

## Data Availability Statement

The datasets analyzed in this article are not publicly available. Requests to access the datasets should be directed to asharma@pennstatehealth.psu.edu.

## Ethics Statement

The studies involving human participants were reviewed and approved by Institutional Review Board (the Penn State University). The patients/participants provided their written informed consent to participate in this study. The animal study was reviewed and approved by Institutional Animal Care and Use Committee (the Penn State University).

## Author Contributions

CA: investigation, conceptualization, design, methodology, formal analysis, visualization, writing-original draft preparation, review, and editing of the manuscript. S-FT, ST, and PD: writing-original draft preparation, review, and revision of the manuscript, investigation. PB, VS, JS, ZZ, and WD: investigation and proofreading. HZ: acquired patient materials, and review of the manuscript. DF and SD: review and editing of the manuscript. TL: funding acquisition, advised and review and editing of the manuscript. SR, AKS, DD, and SA: synthesized and provided the drug. DC: acquired patient materials, supervision, funding acquisition, review, and editing of the manuscript. AS: supervision, investigation, conceptualization, project administration, funding acquisition, design, validation, methodology, formal analysis, writing-original draft preparation, review, and editing of the manuscript.

### Conflict of Interest

TL is on the Scientific Advisory Board and has stock options for both Keystone Nano and Bioniz Therapeutics. The remaining authors declare that the research was conducted in the absence of any commercial or financial relationships that could be construed as a potential conflict of interest.
