# Supplementary material for: The PI3K/AKT Pathway Inhibitor ISC-4 Induces Apoptosis and Inhibits Growth of Leukemia in Preclinical Models of Acute Myeloid Leukemia
Source: Front Oncol. 2020 Apr 1;10:393. doi: 10.3389/fonc.2020.00393 (PMC7140985; doi:10.3389/fonc.2020.00393)
Supplement: Supplementary file 1 [file Presentation_1.pptx]

## Slide 1
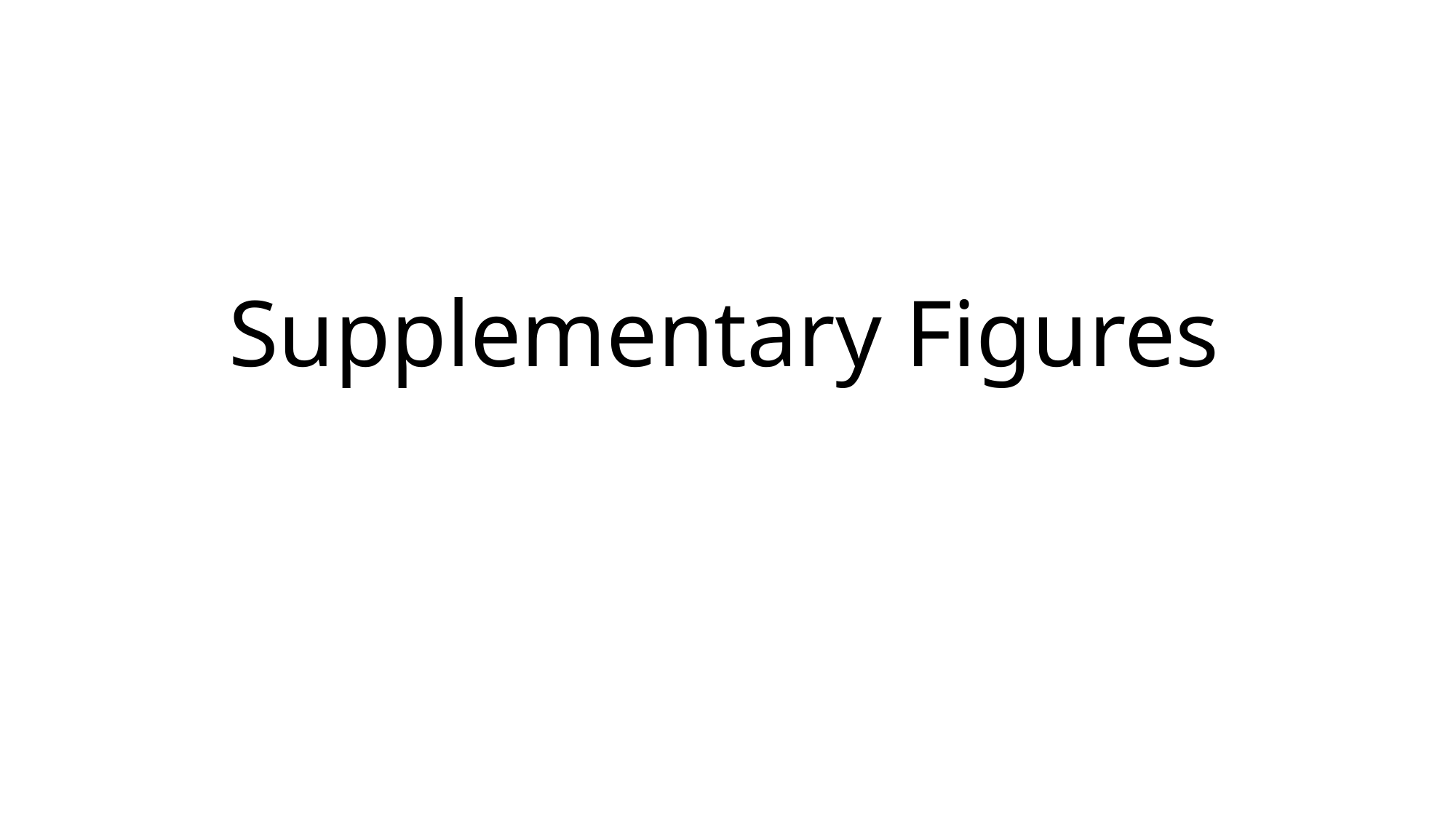

# Supplementary Figures

## Slide 2
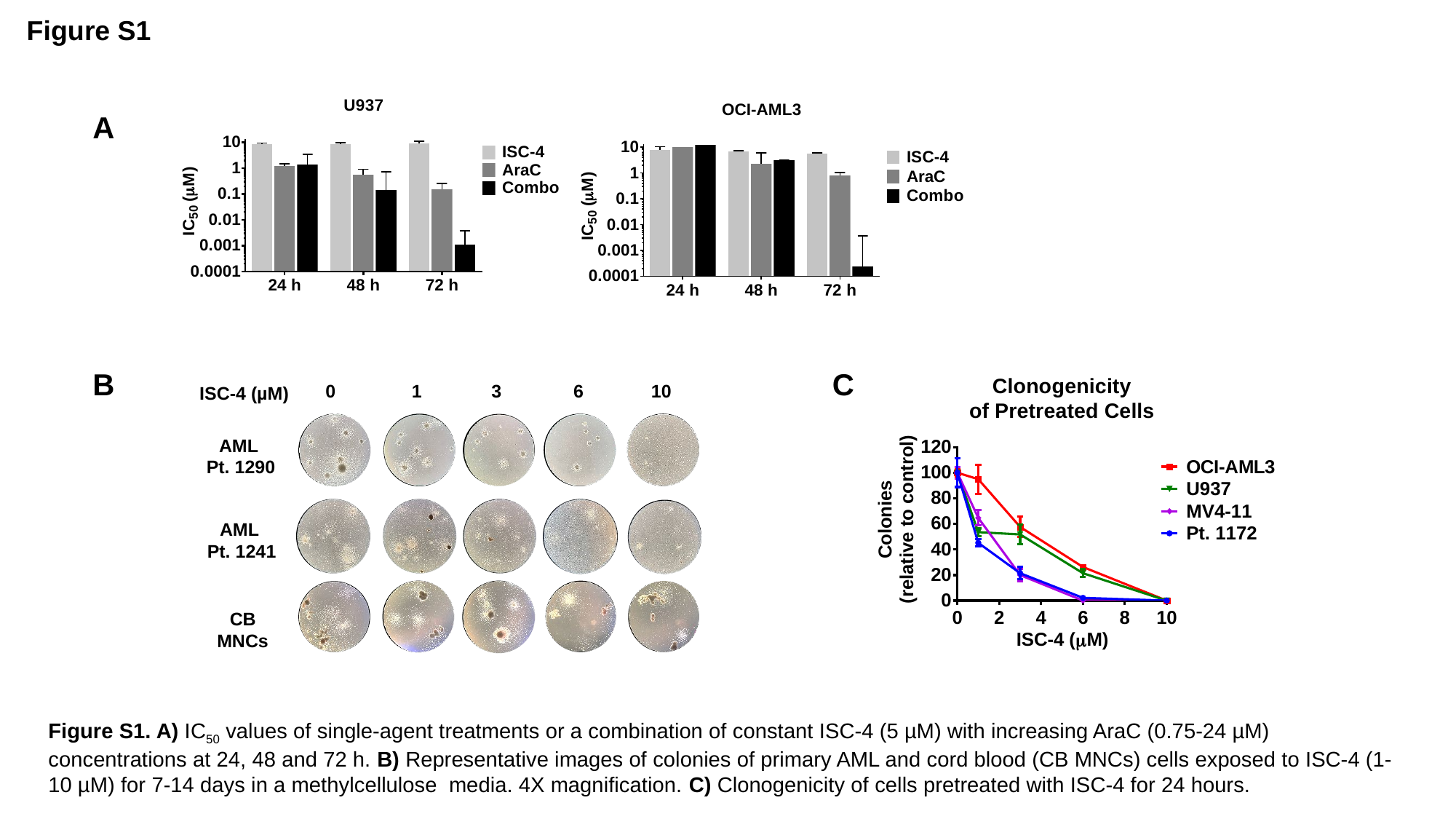

Figure S1
A
B
C
0
1
3
6
10
AML
Pt. 1290
AML
Pt. 1241
CB MNCs
ISC-4 (µM)
Figure S1. A) IC50 values of single-agent treatments or a combination of constant ISC-4 (5 µM) with increasing AraC (0.75-24 µM) concentrations at 24, 48 and 72 h. B) Representative images of colonies of primary AML and cord blood (CB MNCs) cells exposed to ISC-4 (1-10 µM) for 7-14 days in a methylcellulose media. 4X magnification. C) Clonogenicity of cells pretreated with ISC-4 for 24 hours.

## Slide 3
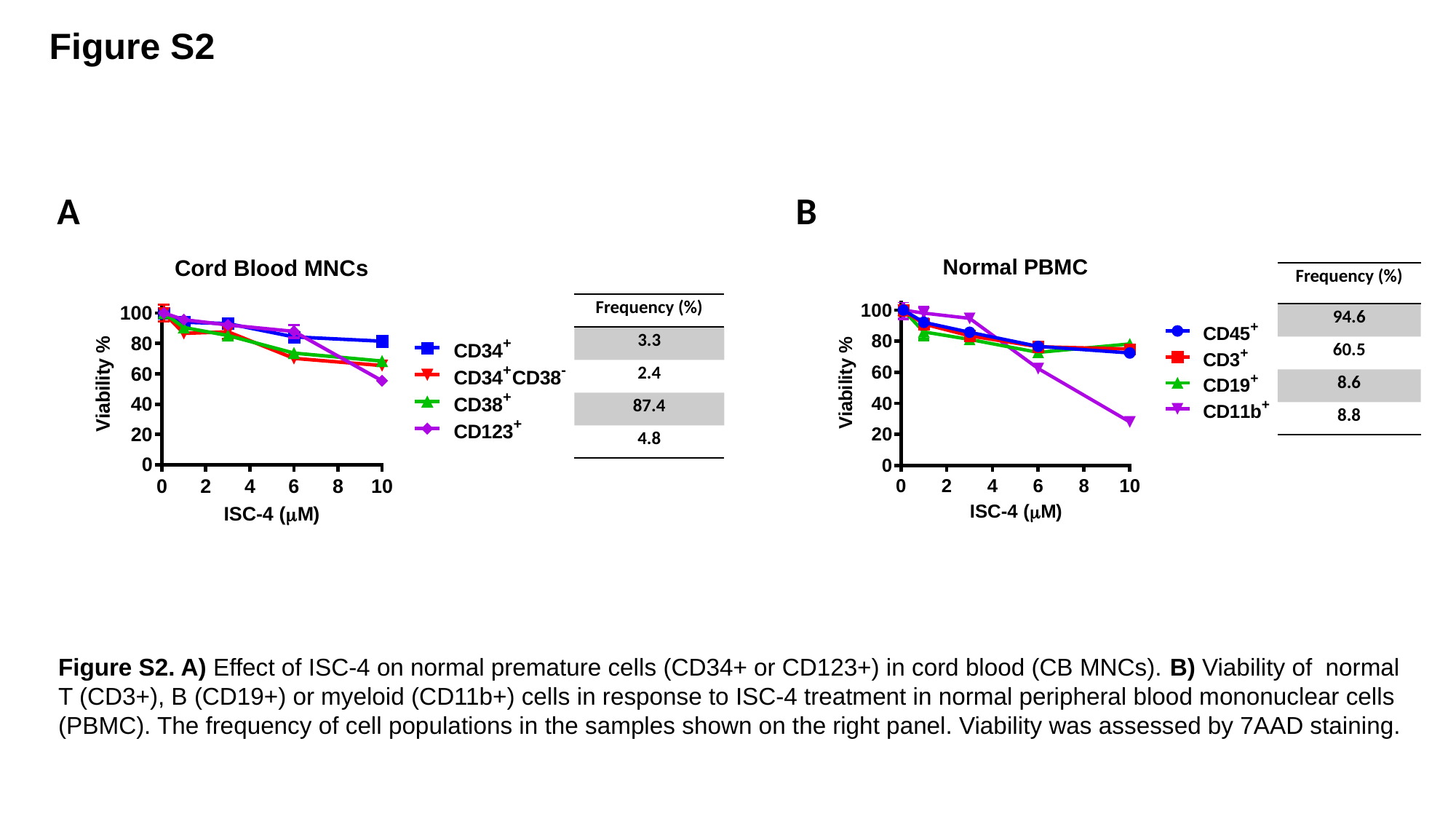

Figure S2
A
B
| Frequency (%) |
| --- |
| 94.6 |
| 60.5 |
| 8.6 |
| 8.8 |
| Frequency (%) |
| --- |
| 3.3 |
| 2.4 |
| 87.4 |
| 4.8 |
Figure S2. A) Effect of ISC-4 on normal premature cells (CD34+ or CD123+) in cord blood (CB MNCs). B) Viability of normal T (CD3+), B (CD19+) or myeloid (CD11b+) cells in response to ISC-4 treatment in normal peripheral blood mononuclear cells (PBMC). The frequency of cell populations in the samples shown on the right panel. Viability was assessed by 7AAD staining.

## Slide 4
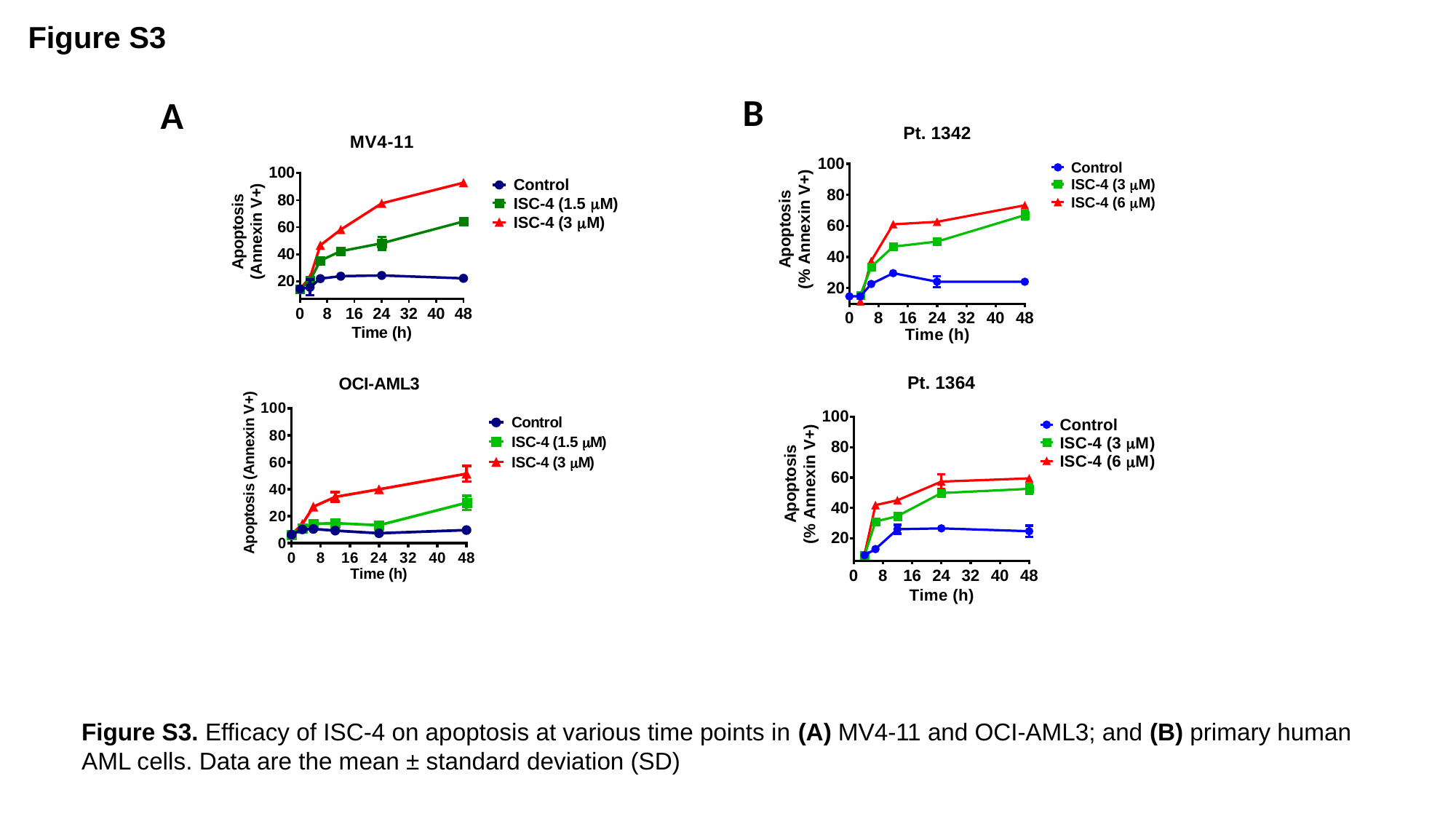

Figure S3
B
A
Figure S3. Efficacy of ISC-4 on apoptosis at various time points in (A) MV4-11 and OCI-AML3; and (B) primary human AML cells. Data are the mean ± standard deviation (SD)

## Slide 5
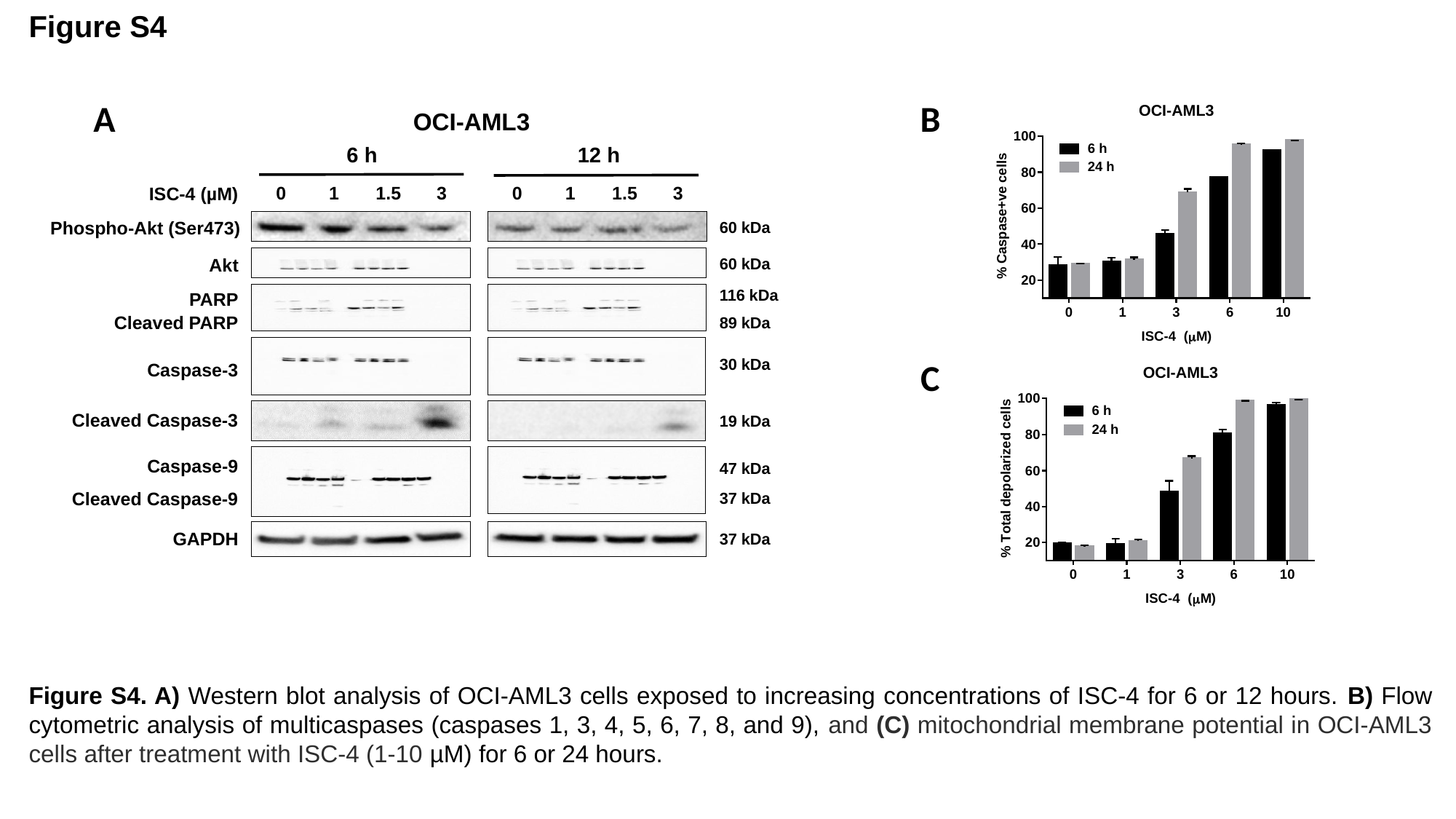

Figure S4
A
B
OCI-AML3
6 h
12 h
3
1
0
1.5
3
1
0
1.5
ISC-4 (µM)
Phospho-Akt (Ser473)
60 kDa
60 kDa
116 kDa
89 kDa
30 kDa
19 kDa
47 kDa
37 kDa
37 kDa
Akt
PARP
Cleaved PARP
Caspase-3
Cleaved Caspase-3
Caspase-9
Cleaved Caspase-9
GAPDH
C
Figure S4. A) Western blot analysis of OCI-AML3 cells exposed to increasing concentrations of ISC-4 for 6 or 12 hours. B) Flow cytometric analysis of multicaspases (caspases 1, 3, 4, 5, 6, 7, 8, and 9), and (C) mitochondrial membrane potential in OCI-AML3 cells after treatment with ISC-4 (1-10 µM) for 6 or 24 hours.

## Slide 6
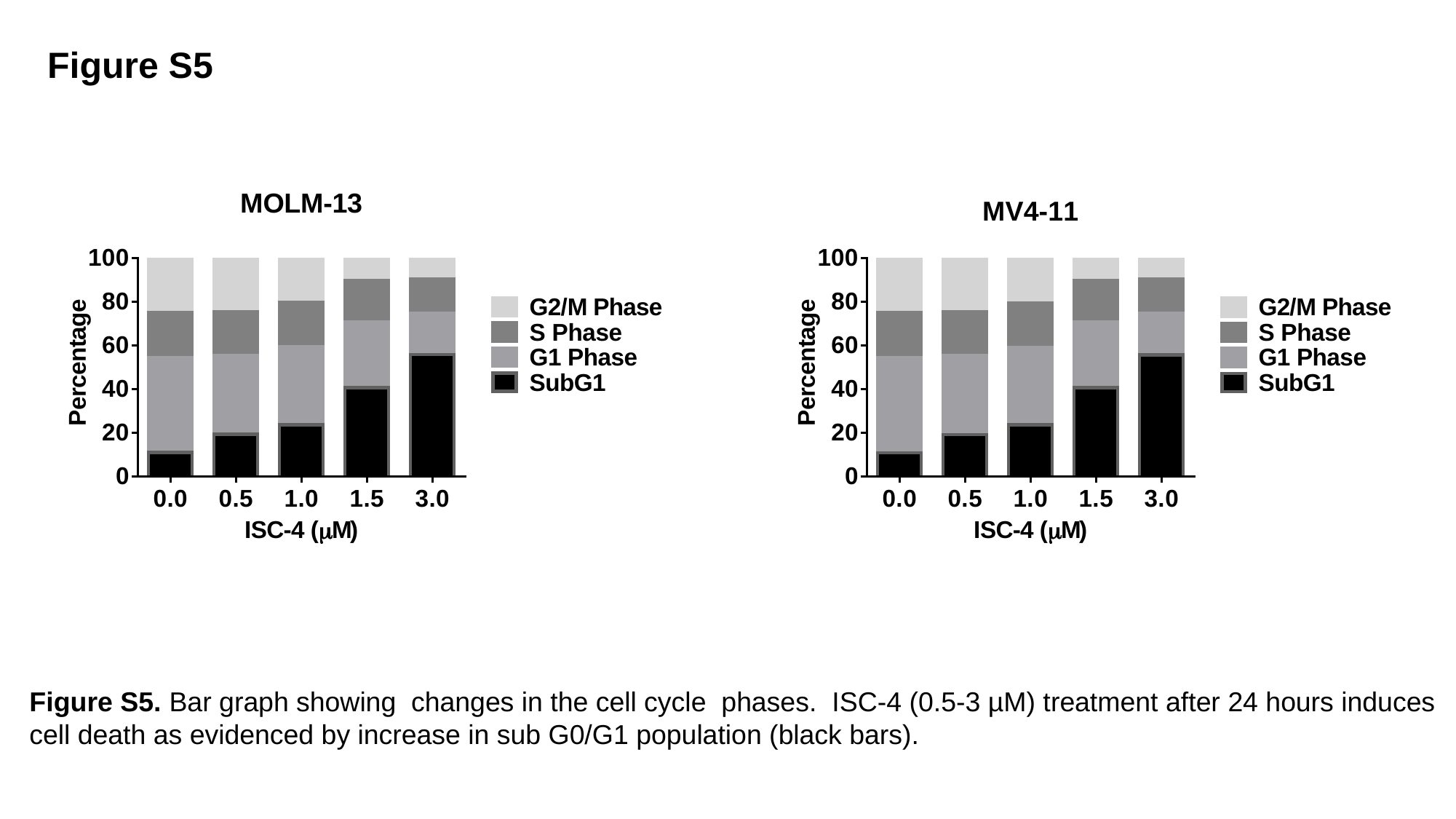

Figure S5
Figure S5. Bar graph showing changes in the cell cycle phases. ISC-4 (0.5-3 µM) treatment after 24 hours induces cell death as evidenced by increase in sub G0/G1 population (black bars).

## Slide 7
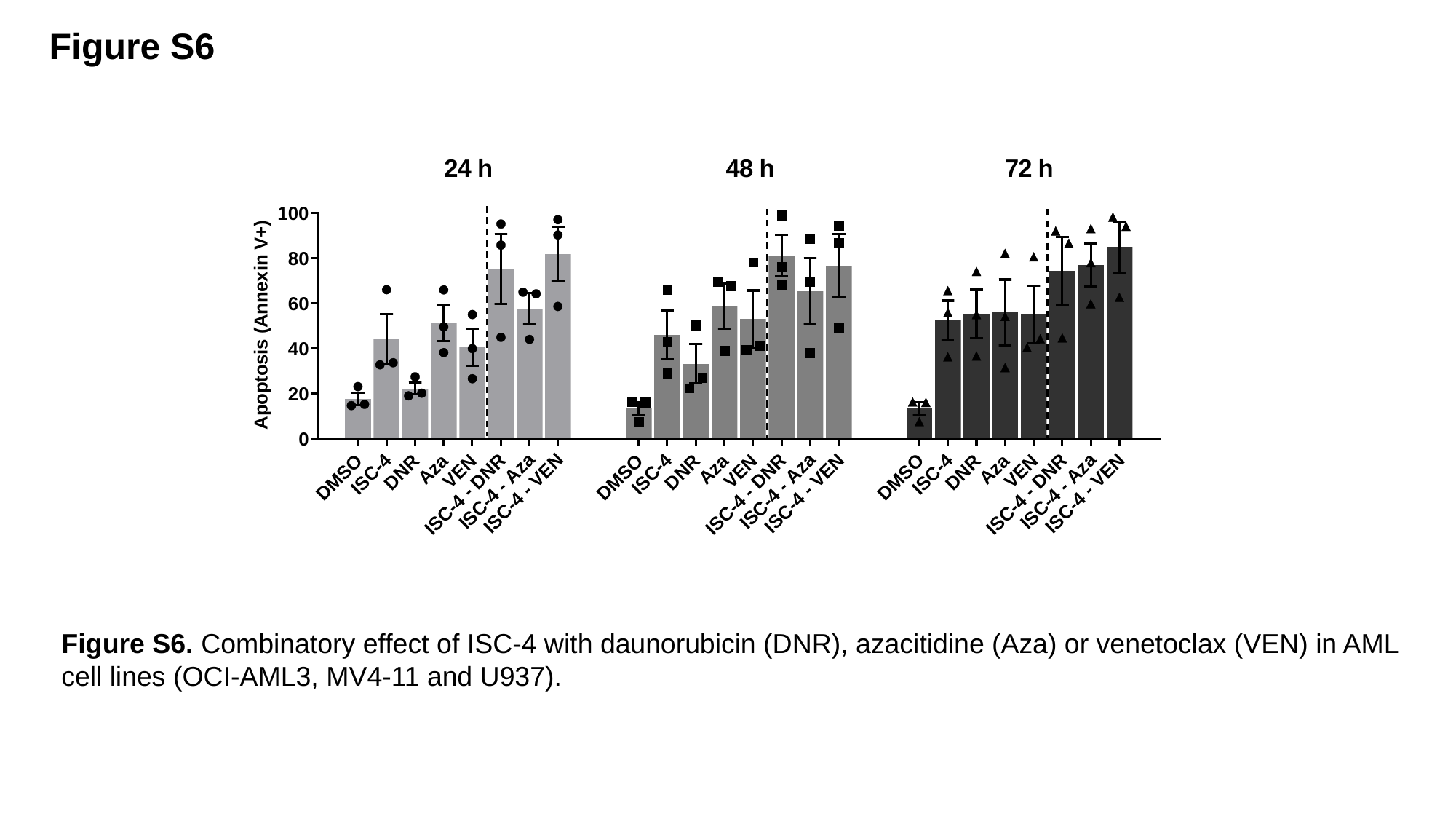

Figure S6
Figure S6. Combinatory effect of ISC-4 with daunorubicin (DNR), azacitidine (Aza) or venetoclax (VEN) in AML cell lines (OCI-AML3, MV4-11 and U937).

## Slide 8
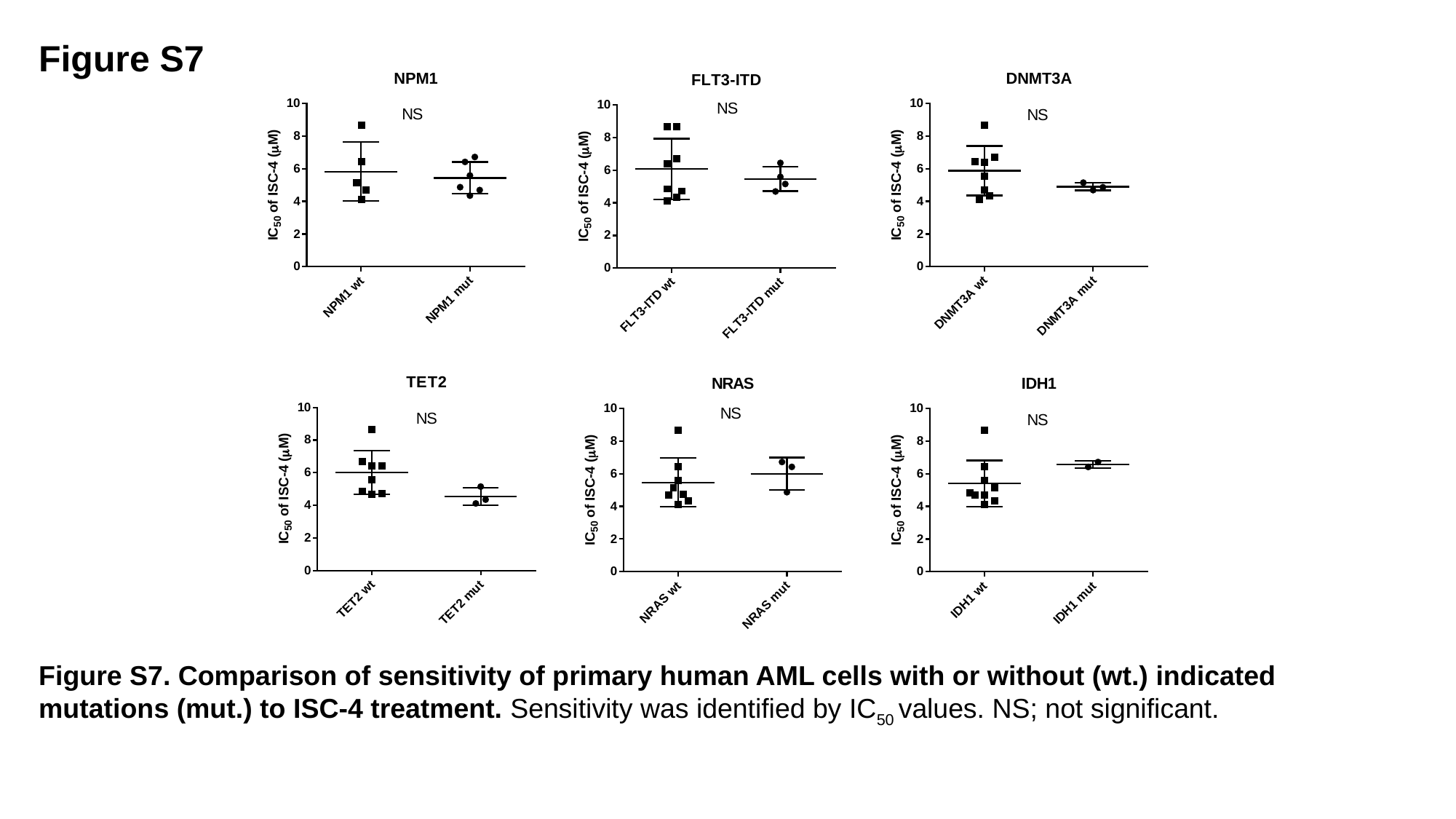

Figure S7
Figure S7. Comparison of sensitivity of primary human AML cells with or without (wt.) indicated mutations (mut.) to ISC-4 treatment. Sensitivity was identified by IC50 values. NS; not significant.

## Slide 9
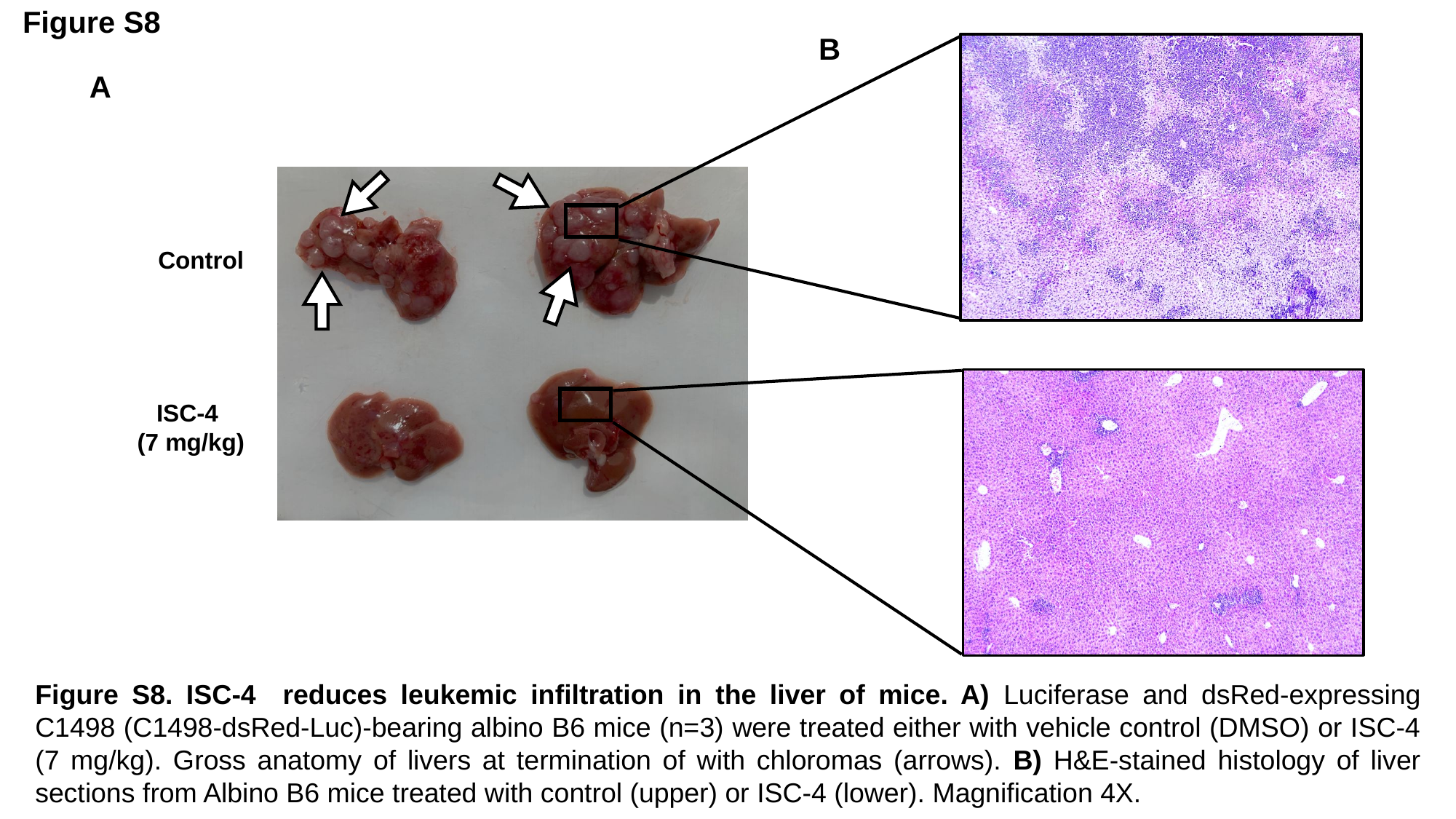

Figure S8
B
Control
ISC-4
(7 mg/kg)
A
Figure S8. ISC-4 reduces leukemic infiltration in the liver of mice. A) Luciferase and dsRed-expressing C1498 (C1498-dsRed-Luc)-bearing albino B6 mice (n=3) were treated either with vehicle control (DMSO) or ISC-4 (7 mg/kg). Gross anatomy of livers at termination of with chloromas (arrows). B) H&E-stained histology of liver sections from Albino B6 mice treated with control (upper) or ISC-4 (lower). Magnification 4X.

## Slide 10
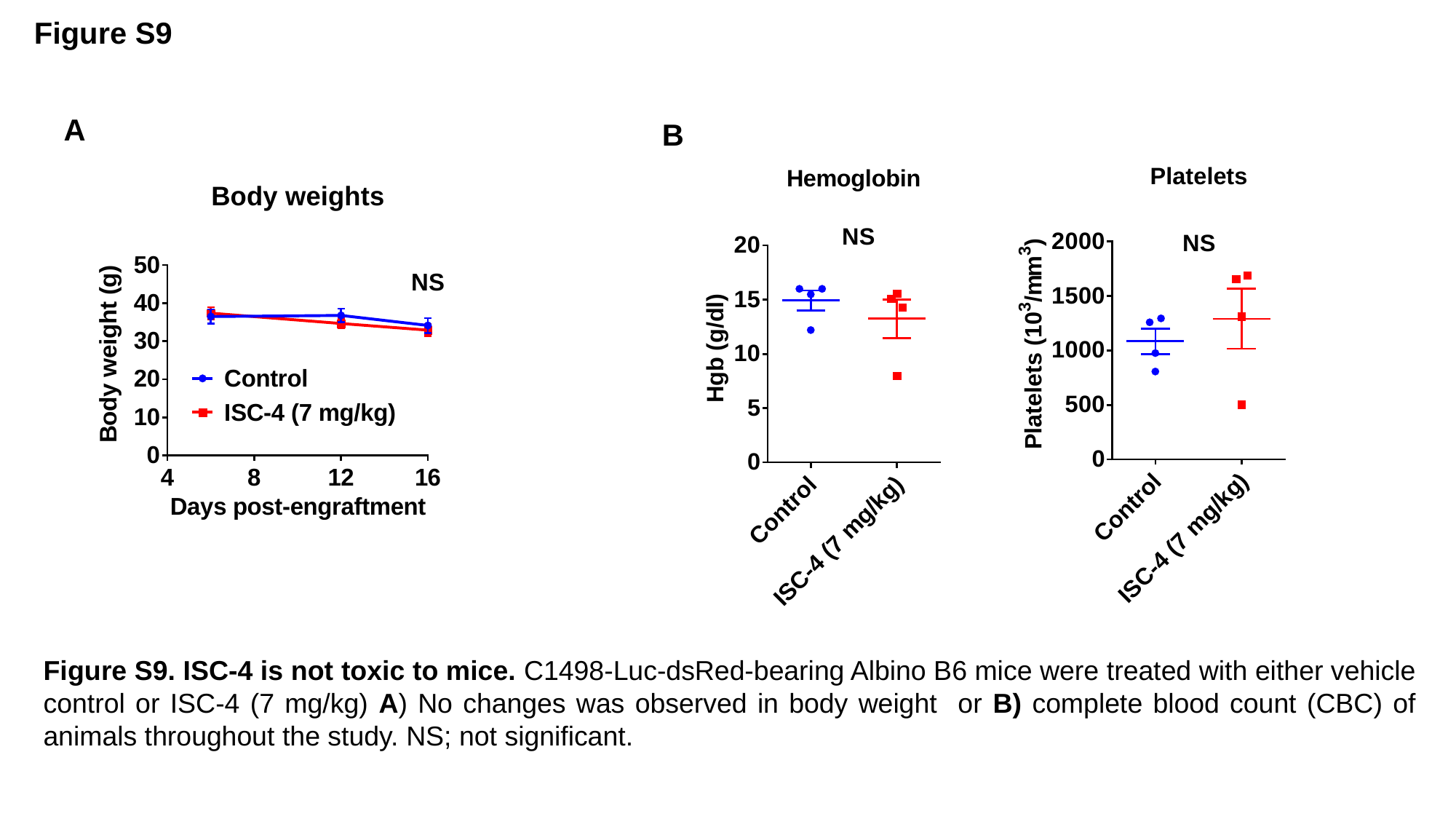

Figure S9
A
B
Figure S9. ISC-4 is not toxic to mice. C1498-Luc-dsRed-bearing Albino B6 mice were treated with either vehicle control or ISC-4 (7 mg/kg) A) No changes was observed in body weight or B) complete blood count (CBC) of animals throughout the study. NS; not significant.
